# Significant role of secondary electrons in the formation of a multi-body chemical species spur produced by water radiolysis

**DOI:** 10.1038/s41598-024-76481-z

**Published:** 2024-10-21

**Authors:** Takeshi Kai, Tomohiro Toigawa, Yusuke Matsuya, Yuho Hirata, Tomoya Tezuka, Hidetsugu Tsuchida, Akinari Yokoya

**Affiliations:** 1 Nuclear Science and Engineering Center, Japan Atomic Energy Agency, 2-4 Shirane Shirakata, Tokai-mura, Naka-gun, Ibaraki, 319-1195 Japan; 2 Faculty of Health Sciences, Hokkaido University, Kita-12 Nishi-5, Kita-ku, Sapporo, 060- 0812 Hokkaido Japan; 3 Department of Nuclear Engineering, Kyoto University, Nishikyo-ku, Kyoto, 615-8530 Japan; 4 Quantum Science and Engineering Center, Kyoto University, Gokasho, Kyoto, 611-0011 Uji Japan; 5 Institute for Quantum Life Science, National Institutes for Quantum Science and Technology, 4-9-1 Anagawa, Inage-ku, Chiba-shi, 263-8555 Japan

**Keywords:** Biophysics, Chemistry, Physics

## Abstract

Scientific insights into water photolysis and radiolysis are essential for estimating the direct and indirect effects of deoxyribonucleic acid (DNA) damage. Secondary electrons from radiolysis intricately associated with both effects. In our previous paper, we simulated the femtosecond (1 × 10^− 15^ s) dynamics of secondary electrons ejected by energy depositions of 11−19 eV into water via high-energy electron transport using a time-dependent simulation code. The results contribute to the understanding of simple “intra-spur” chemical reactions of tree-body chemical species (hydrated electrons, hydronium ion and OH radical) in subsequent chemical processes. Herein, we simulate the dynamics of the electrons ejected by energy depositions of 20−30 eV. The present results contribute to the understanding of complex “inter-spur” chemical reactions of the multi-body chemical species as well as for the formation of complex DNA damage with redox site and strand break on DNA. The simulation results present the earliest formation mechanism of an unclear multi-body chemical species spur when secondary electrons induce further ionisations or electronic excitations. The formation involves electron–water collisions, i.e. ionisation, electronic excitation, molecular excitation and elastic scattering. Our simulation results indicate that (1) most secondary electrons delocalise to ~ 12 nm, and multiple collisions are sometimes induced in a water molecule at 22 eV deposition energy. (2) The secondary electrons begin to induce diffuse band excitation of water around a few nm from the initial energy deposition site and delocalise to ~ 8 nm at deposition energies ~ 25 eV. (3) The secondary electron can cause one additional ionisation or electronic excitation at deposition energies > 30 eV, forming a multi-body chemical species spur. Thus, we propose that the type and density of chemical species produced by water radiolysis strongly depend on the deposition energy. From our results, we discuss formation of complex DNA damage.

## Introduction

Fundamental studies on water photolysis and radiolysis are essential for understanding deoxyribonucleic acid (DNA) damage in radiation biological effects, because the human body mostly comprises water. These scientific insights are particularly valuable for analysing the formation of clustered or multiple DNA damage, which is believed to induce biological effects, such as cell death, mutation induction or carcinogenesis^1–3^. The complexity of DNA damage clustering via direct and indirect effects depends on the density of radiolytic chemical species produced by energy deposited around DNA. In addition to the direct and indirect effects, previous studies reported that shock wave generated by ion irradiation of a living system was also involved in DNA damage^4,5^. The direct effect indicates that DNA damage results from energy deposited by primary radiation and ionised secondary electrons via atomic interactions, such as ionisation or electronic excitation. Meanwhilst, the indirect effect indicates that DNA damage results from the chemical reaction via thermal diffusion of the chemical species (e.g., hydroxyl radical (^•^OH) and hydrated electron (e^−^ _aq_)) produced by the radiolysis of a living system. Therefore, it is essential to investigate the correlation between energy deposition and radiolytic chemical species.

Experimental photolysis and pulse radiolysis techniques have been developed to measure the fast reactions of chemical species resulting from laser or high-energy electron irradiation in the picosecond (ps) order^6–18^. Monochromatic short-pulse lasers allowed the measurement of ultrafast phenomena on the femtosecond (fs) order. These kind of experiments have advanced studies on the formation of pre-hydrated electron (e^−^ _pre_) and e^−^ _aq_ formations^19–23^. In recent years, combining liquid jet and mass spectrometry has also made it possible to measure the cation and anion yields from heavy ion irradiation in aqueous solutions^24^. Although advances in these experimental techniques have led to the accumulation of scientific data for various chemical species, the inhomogeneous spatial distributions of the initial chemical species remain controversial.

When water is irradiated with a high-energy electron, ionisations and electronic excitations considerably occur, inducing numerous single spurs inhomogeneously distributed along the electron tracks in the water. When ionisation is induced, a single spur primarily comprises three chemical species (^•^OH, hydronium ion (H_3_O^+^) and e^−^ _aq_). In this study, we noted that the track indicates the long-distance trajectory of the incident electron and the spur indicates the spatial distribution with nanometer size of the secondary electrons produced by ionisation. In the period up to 100 ps, these chemical species in the spur react with each other (intra-spur reaction). Further down to the nanosecond (ns) order, chemical reactions between the expanding single spurs (inter-spur reaction) are induced within 100 ns, and the concentrations of the chemical species become spatially homogeneous^9^. These timescales depend on the chemical species’ diffusion coefficients and reaction rates and the spur radius and inter-spur distance. Even with modern measurement techniques, it is challenging to measure the spur radius and inter-spur distance by experiments directly.

To solve the scientific issues, the inter-spur distances have been simulated using the Monte Carlo code (MCC)^1,25–34^ (e.g. Kyushu University Radiobiology Unit Code (KURBUC)^1,28,29^, TRACk structure of Electrons in Liquid water (TRACEL)^26^, TRACELE^27^, RITRACKS^30^, PARTRAC^31^, Geant4-DNA^32^ and Particle and Heavy Ion Transport code System (PHITS)^33,34^). Issues remain regarding the spur radius, which requires calculations for electron thermalisation until a few 100 fs and electron delocalisation around 10 nm. Recently, Geant4-DNA and PHITS enabled the electron deceleration calculation down to a few eV. However, conventional MCCs typically set the cut-off energy for electron deceleration at 7−10 eV^35–37^, making the evaluation of the spar radius challenging. Therefore, the spur radius must be modelled based on the experimental results of photo-ionisation^36–39^.

Therefore, Therefore, we developed a dynamic MCC for the physical process (dmcc_phys) consisting of Monte-Carlo (MC), molecular dynamics (MD) methods and database for electron impact cross sections that allows the calculation of the spur radius and inter-spur distances using a time-dependent simulation^40–48^. This code does not require setting the electron cut-off energies. We qualitatively revealed the electron deceleration delocalisation–relocalisation and hydration mechanisms resulting from a 12.4 eV energy deposition on water^47^ and indicated that our estimated spur radius correlates with the experimentally based estimation value^16,47^. We further studied three-body single spurs (^•^OH, H_3_O^+^ and e^−^ _aq_) induced by deposition energies of 11−19 eV^48^. In a living cell, chemical reactions via the chemical spices will form many isolated DNA lesions, which can be easily repaired. However, clustered DNA damage, which contains multiple lesions within 10 base pairs (i.e., 3.4 nm), will slightly be formed via the chemical species of the multi-body chemical species spur, which comprises more than three chemical species (i.e., ^•^OH, H_3_O^+^ and e^−^ _aq_). Advanced knowledge of multi-body chemical species spur formation is needed when applying it to biological effects because clustered DNA damage strongly inhibits enzymatic repair^49–53^.

**Fig. 1:**
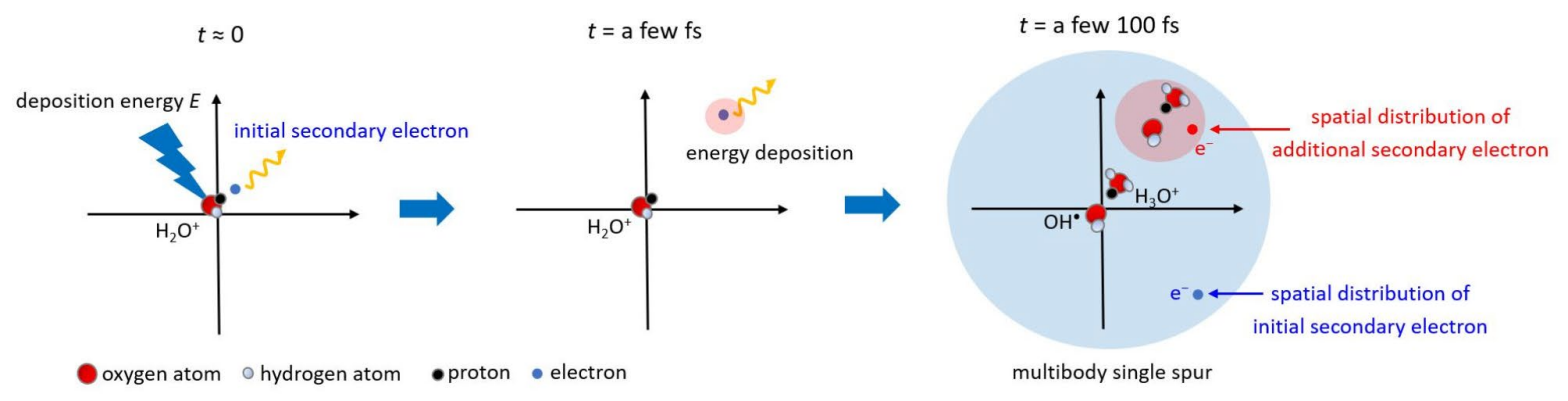
Illustration of multi-body chemical spices spur focused in this study.

This study investigates the fs dynamics of secondary electrons produced by 20−30 eV deposition energy to clarify the earliest formation process of multi-body chemical species spurs using dmcc_phys. This study focuses on the dynamical simulation of only secondary electrons from an ionisation site (Fig. 1). We show the simulation results of the electron spatial distributions, collision frequency distributions in the spurs and the energy dependence of the mean collision frequency and spur radius. These results provide significant insights for interpreting the mechanisms of clustered DNA damage induction.

## Methods

First, we briefly describe the features of our code. We proposed the physical code, dmcc_phys, in 2014^40^. In the earliest development, we used electron impact cross-sections for gas-phase water^40^. In 2015, we reported molecular excitation cross-sections for liquid-phase water^41^ and updated ionisation and electronic excitation cross-sections for liquid-phase water^42^. We successfully demonstrated electron deceleration in extremely low-energy regions on the order of 10^− 3^ eV^42^, showing that the electron energy distributions asymptotically approach the Maxwellian (300 K bulk water) using momentum transfer and molecular excitation cross-Sects^42,47,48^. Using the dmcc_phys, we proposed a new formation mechanism for DNA damage involving e^−^ _pre_.^43–45^ In the study^45^, we indicated a possibility that the clustered DNA damage generated by the mechanism cannot be removed.

Before 2018, we generated secondary electrons at the site where ionisations (1b_1_, 3a_1_, 1b_2_, 2a_1_ and 1a_1_) occur^45^. The latest dmcc_phys enables the simulation of electron dynamics induced from ionisations and electronic excitations (A^1^B_1_, B^1^A_1_, Rydberg (A + B), Rydberg (C + D), diffuse band and collective excitations)^46^. Furthermore, the dielectric response of water was calculated using Fourier transformation of the complex dielectric function of water^46,47^. These improvements led to successful calculations of the initial e^−^ _aq_ yield for the water radiolysis^46^ and the electron spatial distribution for the water photolysis^47^.

We facilitate understanding this calculation method by briefly describing the differences between the conventional MCC and dmcc_phys. The MCC follows the time-independent Monte Carlo (MC) method to simulate electron motion in water^25–37^. Whereas the electron cut-off energy of arbitrary constant must typically be set in the MCC^28,35^, dmcc_phys follows the time-dependent MC and molecular dynamics (MD) methods to output the spatial and energy distributions of secondary electrons at each time^42–48^. Therefore, it is necessary to set the cut-off time in our dmcc_phys. The cutoff time is the time when the energy distribution of secondary electrons is close to Maxwellian of 300 K^46–48^. Our code can simulate the dynamical and collisional motions of the secondary electrons in the long-range Coulombic field created by the parent cation from moment to moment. The shielding of this Coulombic field along the dielectric response simulates the hydration process^46–48^. Especially, this hydration model revealed time evolution with the deceleration, thermalisation, precursor-hydration and hydration of electrons generated by the water photolysis^47^. The cross-section, time-dependent MC and MD methods, simulation setup and flowchart of our code are briefly described in the section below.

**Fig. 2:**
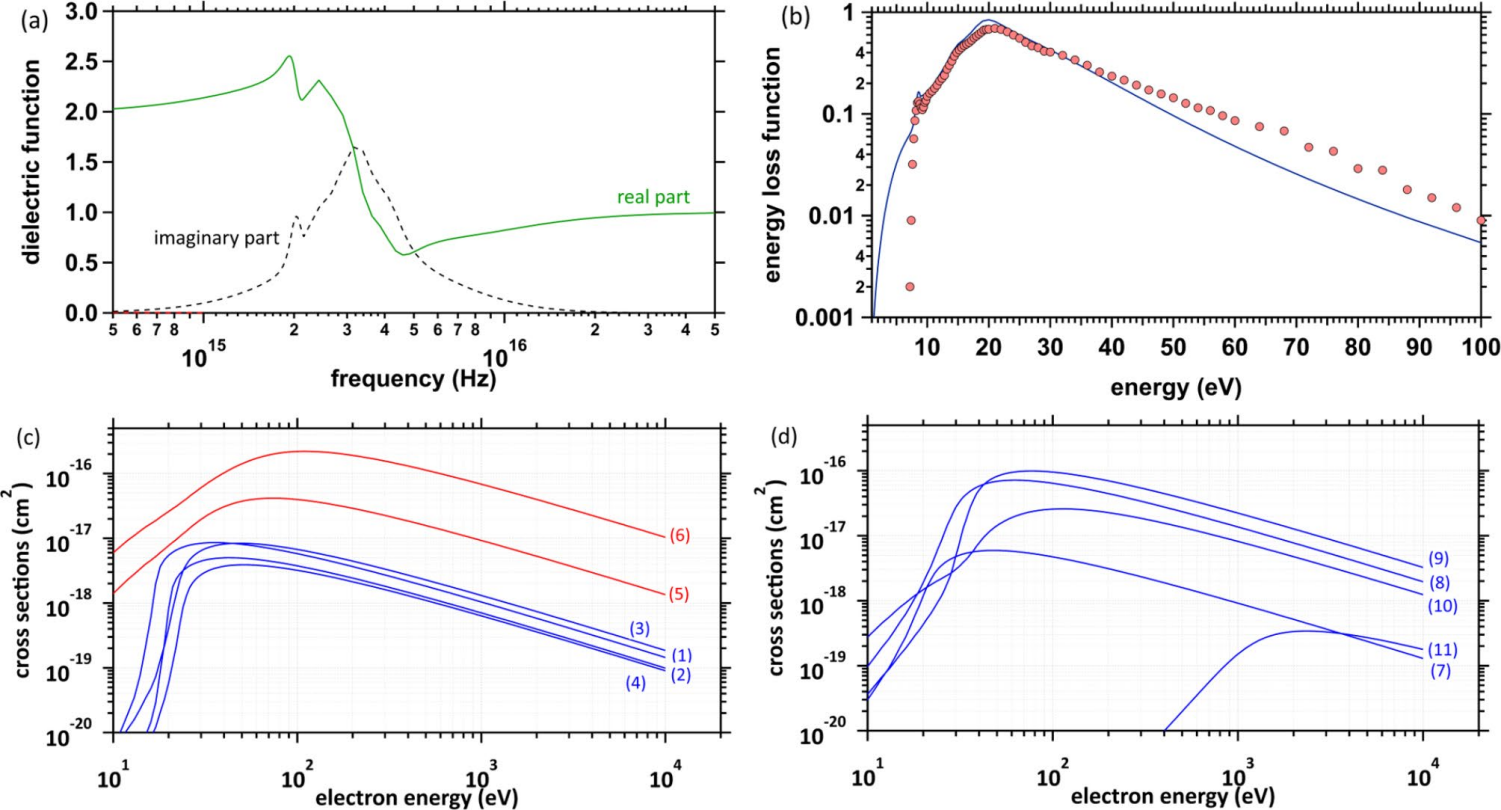
(**a**) Dielectric function calculated using previously reported fitting parameters^26^. (**b**) Energy loss function (ELF) calculated using previously reported fitting parameters (bark blue)^26^. ELF measured by Hayashi et al. (red circles)^58^. (**c**) Electronic excitation cross-sections: (1) A^1^B_1_ excitation (8.4 eV); (2) B^1^A_1_ excitation (10.1 eV); (3) Rydberg (A + B) excitation (11.25 eV); (4) Rydberg (C + D) excitation (11.93 eV); (5) diffuse band excitation (14.1 eV); (6) collective excitation (21.4 eV). The energy in parentheses represents the mean value of the transition energy^56^. (**d**) Ionisation cross-sections: (7) 1b_1_ ionisation (10.9 eV); (8) 3a_1_ ionisation (13.5 eV); (9) 1b_2_ ionisation (17.0 eV); (10) 2a_1_ ionisation (26.3 eV); (11) 1a_1_ ionisation (533 eV). The energy in parentheses represents the mean value of the transition energy^57^. The collective excitation depicted as (6) in Fig. 2c, which is the highest cross-section, was assigned by Paretzke et al.^56^. , however, physical interpretation for the collective excitation is controversial, in general, not widely accepted.

### Cross-sections

The inelastic–scattering cross-sections of liquid water can be calculated using the energy loss function (ELF), obtained from the complex dielectric function^54^. Experimental results of the complex dielectric function of water^55^ and the fitting parameters of the complex dielectric function^26^ have been reported. Figure 2a and b show the complex dielectric function and ELF results, respectively. The ELF represents the energy absorption efficiency of water, with a maximum of approximately 21.4 eV^55^. The absorption efficiency decreases monotonically for the high-energy region above 22 eV in Fig. 2b. First, we present electronic excitation and ionisation cross-sections. The cross-sections *σ* were calculated using *σ* = 1/(*λN*), where *λ* and *N* are the mean free path and molecular density (3.318565 × 10^22^ molecules/cm^3^), respectively. Each ionisation and electronic excitation level was assigned according to two literature^56,57^. We used 11 fitting parameter sets of the complex dielectric function, *ε*(0,*ω*’), as a function of energy transfer *ω*’^26^, and calculated each inverse mean free path *λ*^−1^ from Ashley’s formula^54^,

1 $$\:{\lambda\:}^{-1}\left(E\right)=\frac{1}{2\pi\:E}{\int\:}_{0}^{\raisebox{1ex}{$E$}\!\left/\:\!\raisebox{-1ex}{$2$}\right.}d{\omega\:}^{{\prime\:}}\text{I}\text{m}\left(\frac{-1}{\epsilon\:\left(0,{\omega\:}^{{\prime\:}}\right)}\right)L\left(\frac{{\omega\:}^{{\prime\:}}}{E}\right)\text{d}{\omega\:}^{{\prime\:}},$$

where

$$\:L\left(a\right)=\left(1-a\right)\text{l}\text{n}\frac{4}{a}-\frac{7}{4}a+{a}^{\raisebox{1ex}{$3$}\!\left/\:\!\raisebox{-1ex}{$2$}\right.}-\frac{33}{32}{a}^{2},\:\left(a\ll\:1\right),$$

where *E* is the kinetic energy of an electron interacting with liquid water. Figure 2c and d show the electronic excitation and ionisation cross-sections, respectively. Deposition energies of 20−30 eV to water eject secondary electrons with kinetic energies of 10−20 eV when subtracting the ionisation energy (10.9 eV)^57^, resulting in additional collision events by the secondary electrons. The diffuse band and collective excitation cross-sections highlighted in red exceed 1 × 10^− 18^ cm^2^ in the 10–20 eV energy region in Fig. 2c. It is thought that secondary electrons with a few 10 eV can induce these excitations. A further increase in the deposition energy will also induce 3a_1_ and 1b_2_ ionisations, which have relatively high cross-sections (Fig. 2d). We used the complex dielectric function reported by Heller et al.^55^. In 2015, Hayashi et al. also reported the function that may be accurate^58^. We also plotted the data reported by Hayashi et al.^58^. to compare Heller’s data^55^ in Fig. 2b. There is a conspicuous discrepancy particularly in the energy region above 20 eV. This difference will affect the values of the ionization and electronic excitation cross sections. Since the main purpose of this study was not to calculate the cross sections, the calculation would be a future work.

**Fig. 3:**
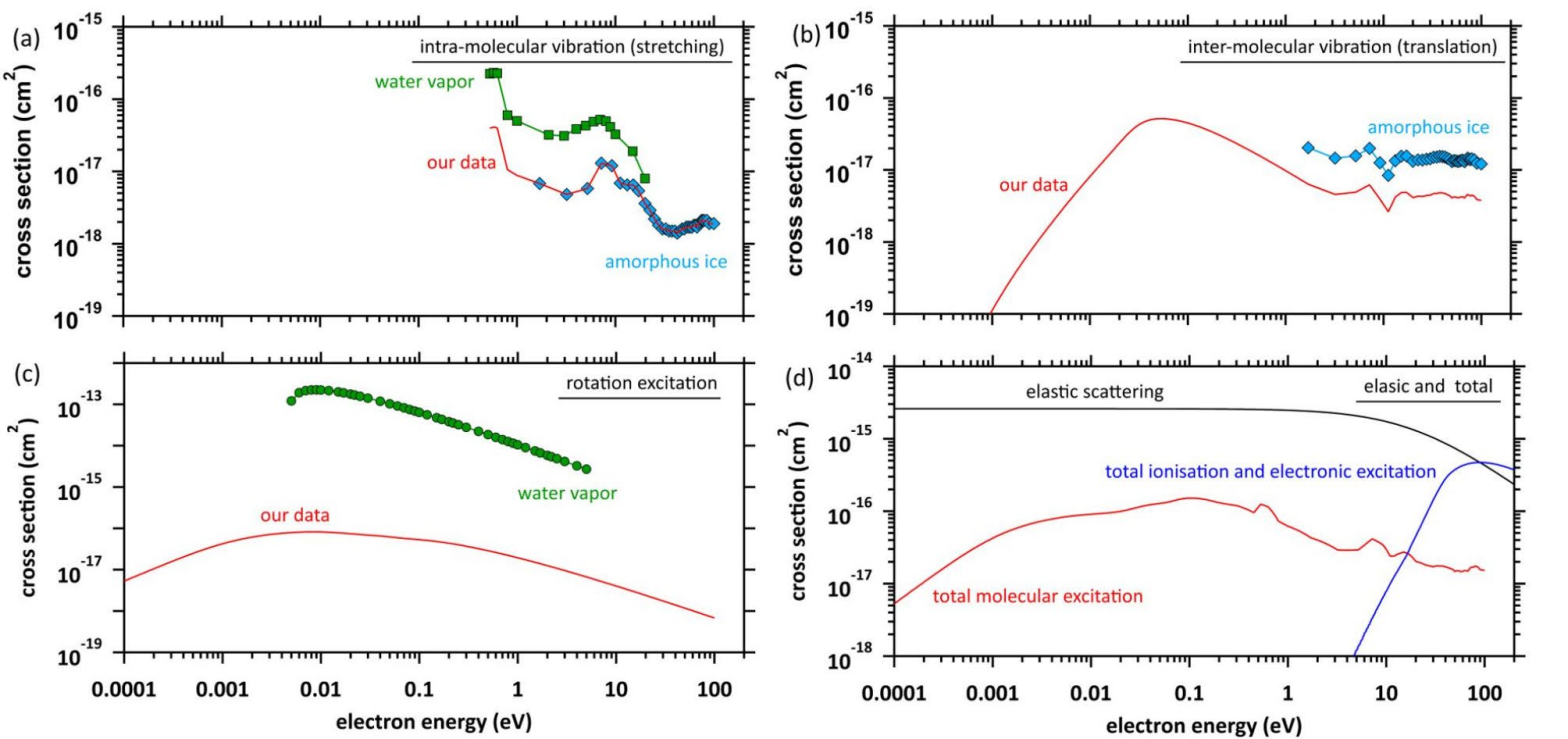
(**a**) Intra-molecular vibration excitation cross-sections, red solid line: our data, ☐: water vapor^59^ ◇: amorphous ice^60^, (**b**) inter-molecular vibration excitation cross-sections, red solid line: our data, ◇: amorphous ice^60^, (**c**) rotation excitation cross-sections, red solid line: our data, 〇: water vapor^59^ and (**d**) red solid line: total molecular excitation cross-section, blue solid line: total ionization and electronic excitation, black solid line: elastic scattering.

The electron impact cross-sections for elastic scattering and molecular excitations are essential for simulating secondary electron motion. Figure 3a shows the intra-molecular vibrational excitation cross-sections. We used the amorphous ice data to obtain the cross-sections of the condensed phase^59^. The data were connected by scaling the water vapour data^60^ to the amorphous ice data because no data below 1.7 eV have been reported. Figure 3b shows the inter-molecular vibration excitation cross-sections. In our previous study^41^, the data were calculated with optical approximation using the ELF of liquid water^61^. However, since the approximation cannot reproduce the resonance structure, the data above 1.7 eV were connected by scaling the amorphous ice data to our data. Figure 3c shows the rotation excitation cross-sections for liquid water. The data were calculated with optical approximation using the ELF of liquid water in our previous study^41^. The rotation excitation cross-section for water vapour^60^ is also shown for comparison. We also show the elastic scattering, total molecular excitation and total ionisation and electronic excitation cross-sections in Fig. 3d.

Moliere’s elastic scattering cross-section was used^62^. The differential and integral cross-sections, *q*(*θ*) and *σ*_elas_, are represented by the following formulas:^62^

2 $$\:q\left(\theta\:\right)=\pi\:Z\left(Z+1\right){r}_{e}^{2}\frac{1-{\beta\:}^{2}}{{\beta\:}^{4}}\frac{1}{{\left(1-\text{cos}\theta\:+2\eta\:\right)}^{2}},$$

and

3 $$\:{\sigma\:}_{\text{e}\text{l}\text{a}\text{s}}=\pi\:Z\left(Z+1\right){r}_{e}^{2}\frac{1-{\beta\:}^{2}}{{\beta\:}^{4}}\frac{1}{\eta\:\left(\eta\:+1\right)},$$

where the screening parameter, $\:\eta\:$, is given by

$$\:\eta\:={\eta\:}_{c}\times\:1.7\times\:{10}^{-5}{Z}^{\raisebox{1ex}{$2$}\!\left/\:\!\raisebox{-1ex}{$3$}\right.}\frac{1}{\tau\:\left(\tau\:+1\right)},$$

where *θ* is the scattering angle, the effective atomic number *Z* of the water molecule was assumed to be 7.42, the classical electron radius *r*_e_ = 2.8179 × 10^− 13^ cm, *β* is the ratio of the velocity of the electron to the velocity of light, *τ* = *E*/*m*_0_*c*^2^ and *E*, *m*_0_ and *c* are the electron energy, electron rest mass, and velocity of light, respectively,

*η*_*c*_ = 1.198 for *E* < 50 keV,

$$\:=\:1.13+3.76{\left(\frac{Z}{137\beta\:}\right)}^{2}$$　*E* > 50 keV.

When elastic scattering is induced, no energy change occurs in the relative motions of an electron and a water molecule; however, the energy for the motion of the centre-of-mass system changes^63,64^. This phenomenon is evaluated using the momentum transfer cross-section, *σ*_mom_, from the differential cross-section, *q*(*θ*), of elastic scattering^63,64^,

4 $$\:{\sigma\:}_{\text{m}\text{o}\text{m}}\:=\:2{\int\:}_{0}^{\pi\:}\left(1-\:\text{cos}\theta\:\right)q\left(\theta\:\right)\text{sin}\theta\:d\theta\:.$$

Using the *σ*_mom_ of Eq. (4) and the integral elastic scattering cross-section *σ*_elas_ of Eq. (3), the energy transfer is given as^46–48,63,64^

5 $$\Delta {\text{E}} \cong \:\frac{{2{\text{~}}m}}{M}\frac{{\sigma \:_{{{\text{mom}}}} }}{{\sigma \:_{{{\text{Elas}}}} }}\left( {E_{{\text{e}}} \: - \:E_{{{\text{mol}}}} } \right) ,$$

where *m* and *M* are the mass and *E*_e_ and *E*_mol_ are the kinetic energies of the electron and water molecules, respectively. *E*_mol_ was sampled from the Maxwellian of 300 K bulk water. When *E*_e_ > *E*_mol_, the electrons provided little energy to the water, whereas when *E*_e_ < *E*_mol_, the electrons received some energy from the water. After the electrons emitted from water molecules are sufficiently decelerated by molecular excitations, the electron energy is mainly determined by the Eq. (5). Therefore, the energy decreases and increases repeatedly by momentum transfer due to elastic scattering between electrons and water until the cut-off time. Thus, our code does not have a specific lower limit in electron energy and provides an energy distribution approaching to a Maxwellian. Finally, the electron kinetic energy distribution approaches the Maxwellian of 300 K bulk water^46–48^, although Δ*E* is approximately a few µeV.

Since the conventional MCC (physical code) cannot simulate electron deceleration processes below a few eV, the spur radius must be modelled based on the experimental results of photo-ionisation^36–39^. In fact, these MCCs correct for the initial position of the e − aq in the physicochemical process^65^. The electron deceleration process is typically categorized as a physicochemical process. Our code consists of the MC and MD methods. Same as the conventional MC codes^25–34^, we also implemented the cross sections shown in Figs. 2 and 3 into our code. This implementation allows for electron deceleration calculation in water (physicochemical process), resulting in connections to chemical processes. The MD method allows for the electron relocalisation calculation to the parent cation^46–48^.

### Time-dependent MC and MD methods

The conventional MCC gives the one-step distance of an electron moving to the next collisional position in the water as Δ*s* = −*λ*ln(*k*); *k* is a uniform random number. Our code assumes that collisions between electrons and water are induced if the following conditions are satisfied:^46–48^

6 $$\:1-\text{exp}\left(-\frac{\varDelta\:s}{\lambda\:}\right)>k,$$

where Δ*s* = *v*Δ*t*, where *v* is the absolute value of the electron velocity, and Δ*t* is the time step set to 1 attosecond (1 × 10^− 18^ s). After the electron collision coordinate is determined, the collision process is identified and sampled from the ratio of each cross-section. From Fig. 3d, the inelastic–scattering cross-sections for the rotation and inter- and intra-molecular vibration modes are close to zero with decreasing electron energy; however, the elastic scattering cross-section has a considerable finite value. Thus, the mean free paths *λ* of extremely low-energy electrons have noticeably short ranges.

When elastic scattering is induced, the scattering angle is sampled from the differential cross-section *q*(*θ*) in Eq. (2). When molecular excitation is induced, it is assumed that the scattering angle does not change since the frequency of molecular excitation is considerably less than that of elastic scattering (Fig. 3d). When ionisation or electronic excitation is induced, the scattering angles *θ*_p_ and *θ*_s_ for primary and secondary electrons are given by the energy and momentum conservation laws, respectively, as shown in Eqs. (7),^26^

7 $$\:{\text{s}\text{i}\text{n}}^{2}{\theta\:}_{p}=\frac{\raisebox{1ex}{${E}_{2}$}\!\left/\:\!\raisebox{-1ex}{${E}_{1}$}\right.}{\left(1-\raisebox{1ex}{${E}_{2}$}\!\left/\:\!\raisebox{-1ex}{${E}_{1}$}\right.\right){E}_{1}\left(2{m}_{0}{c}^{2}\right)+1},\:{\text{s}\text{i}\text{n}}^{2}{\theta\:}_{s}=\frac{1-\raisebox{1ex}{${E}_{2}$}\!\left/\:\!\raisebox{-1ex}{${E}_{1}$}\right.}{1+\raisebox{1ex}{${E}_{2}$}\!\left/\:\!\raisebox{-1ex}{$\left(2{m}_{0}{c}^{2}\right)$}\right.},\:\:\:\:$$

where *E*_1_ is the kinetic energy of the colliding electron, and the initial kinetic energy *E*_2_ of the generated electron is sampled from the ELF (Fig. 2b). The azimuthal angle *φ* is sampled from a uniform random number, determining the post-collision velocity vector.

**Fig. 4:**
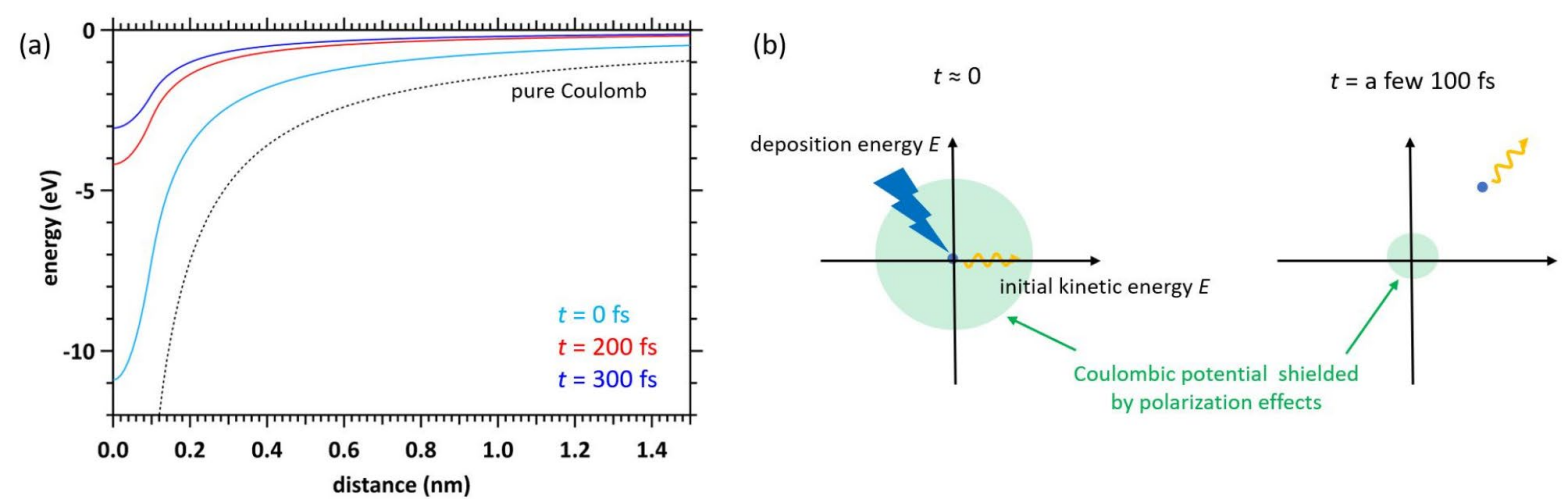
(**a**) Potential energy included in the polarisation effect of the finite-size particle model assumed in this code. (**b**) Time evolution of the potential energy included in the polarisation effect of the finite-size particle model assumed in this code.

We assumed that electrons and cations are finite-size particles of radius *a* with negative and positive charges (finite-size particle model)^46–48^. The particle radius was 0.099 nm to reproduce the lowest ionisation energy of 10.9 eV^57^, and a minimum position of the potential energy (–10.9 eV) was allocated at the origin. When the potential of the cation is expressed in spherical coordinates, it can be expressed as,

8 $$\:\varPhi\:\left(r\right)=\frac{1}{4\pi\:\epsilon\:}{\int\:}_{-\infty\:}^{\infty\:}\frac{e}{\left|\varvec{r}-{\varvec{r}}^{{\prime\:}}\right|}d{\varvec{r}}^{{\prime\:}}=\frac{e}{4\pi\:\epsilon\:r}\:\left(r\ge\:a\right)\:\text{o}\text{r}\:\frac{e\left(3{a}^{2}-{r}^{2}\right)}{8\pi\:\epsilon\:{a}^{3}}\:\left(r<a\right),$$

where *e* is the elementary charge, and *ε* = *ε*_0_ × *ε*_r_(*t*). *ε*_0_ is the dielectric constant of a vacuum and *ε*_r_(*t*) is the dielectric response reported in our previous papers^46,47^. This result causes the potential energy to change with time evolution, as reported previously^46,47^. Figure 4 (a) and (b) show the potential energy and an illustration of potential energy-shielding by the polarisation effect. In conventional MCCs^27–35^, the kinetic energy of the secondary electron is obtained by subtracting the ionisation energy from the deposition energy. However, in our code, a secondary electron begins to move with its deposition energy (20–30 eV) from the position (origin) of a potential minimum. When a new ionisation or electronic excitation occurs, the time of dielectric response is 0 and begins anew. The dynamical and collisional motion of the secondary electron in the dynamical Coulombic field in water can be calculated by solving the Newtonian equation in Eq. (9) and the change in the velocity vector due to collisions in Eqs. (2) and (7):

9 $$\:\frac{d}{dt}\mathbf{x}=\mathbf{v}\:,\:m\frac{d}{dt}\mathbf{v}=\mathbf{F},$$

where

$$\:\mathbf{F}=\frac{{-e}^{2}}{4\pi\:\epsilon\:{r}^{3}}\mathbf{r}\:\left(\left|\mathbf{r}\right|\ge\:a\right)\:\text{o}\text{r}\:\mathbf{F}=\frac{{-e}^{2}}{4\pi\:\epsilon\:{a}^{3}}\mathbf{r}\:\left(\left|\mathbf{r}\right|<a\right),\:$$

where **F** is the force between the electron and the cation, and *m* is the electron mass. The electron coordinate is given by **x**, *r* (= |**r**|) is the relative distance between the electron and the cation, and **v** is the velocity vector of the electron. Chemical species, such as H_3_O^+^ and ^•^OH, are assumed to be immobile on the order of fs.

Charges generated in water are shielded by the polarization effect. The dielectric response which shields the charge is very fast in water^47^, and the electron-water collision considerably delays the recombination time between the electrons and the parent ion. For these reasons, many ionised electrons become delocalized, and some electrons distributed within 1 nm of the parent ionic core can be relocalised during a few 100 fs^46–48^. Initial and additional electrons do not relocalise unless they reach a region within 1 nm of another parent ionic core. The possibility of this process would be not high. Therefore, we considered only the two-body Coulombic interaction between the electron and its parent ion to reduce the simulation cost. Under this assumption, we have successfully estimated the initial yield of e^−^ _aq_ from the ratio of delocalized to relocalised components of ionised electrons generated by a low-density electron irradiation field in our previous study^46^. For much higher-density radiation field, on the other hand, the multi-body Coulombic interaction needs to be taken into account.

### Code validation

 Time-dependent density function theory (TDDFT), following quantum theory, is a powerful tool for studying the electronic states of molecules. However, the number of target molecules is limited to one or two and the computation time is about 10 fs^66^, making it difficult to analyse the spur formation field in which many molecules are involved in the process. Therefore, we have developed our code that is a good analyser handling a space of about 10 nm and a time of about several hundred fs in water. To date, we validated our code to compare experimental data. First, we calculated the thermalisation lengths to compare the experimental data^67^ of low energy electrons ejected by photo-injections into electrode in water^46^. Second, we calculated the spur radius to compare the experimental data^11–16^ for water photolysis^47,48^. In those comparisons, we reproduced the experimental data, validating our MC method. Third, we also estimated the initial yield of e^−^ _aq_ from the ratio of delocalised to relocalised components of ejected electrons in 10 keV electron irradiation to water^46^, validating our MD method which that allows electron relocalisation.

### Simulation setup

In this study, the initial coordinate of deposited energy to water is assumed to be at the origin (0, 0, 0). All deposition energy *E*_dep_ is converted to the kinetic energy *E* of the initial secondary electron at the origin. The initial velocity vector of the electron with the kinetic energy is set in the z-axis direction. The Coulombic potential of the cation immediately slows down the initial secondary electrons ejected from the origin^45^. The secondary electrons induce ionisation and electronic excitations to the surrounding water molecules around parent cations and become thermalised–delocalised and relocalised by elastic scattering and molecular excitations^46–48^. H_3_O^+^ and ^•^OH are at the initial and additional energy deposition sites. The dielectric response gradually shields the Coulombic force of the parent cation^47^. Although this shielding effect becomes stronger as time passes in the order of picoseconds, this Coulombic force might cause electrons to relocalise into parent cations within a few 100 fs^46–48^. When the secondary electrons are relocalised, chemical species, such as H^•^ + ^•^OH rather than H_3_O^+^ + ^•^OH + e^−^ _aq_, are produced at the initial or additional energy deposition sites^17,46–48^. Our simulations were performed based on uniform random numbers in the MC, and the number of calculation trials was adapted to reach a statistical uncertainty of much less than 1%.

### Flowchart

**Fig. 5:**
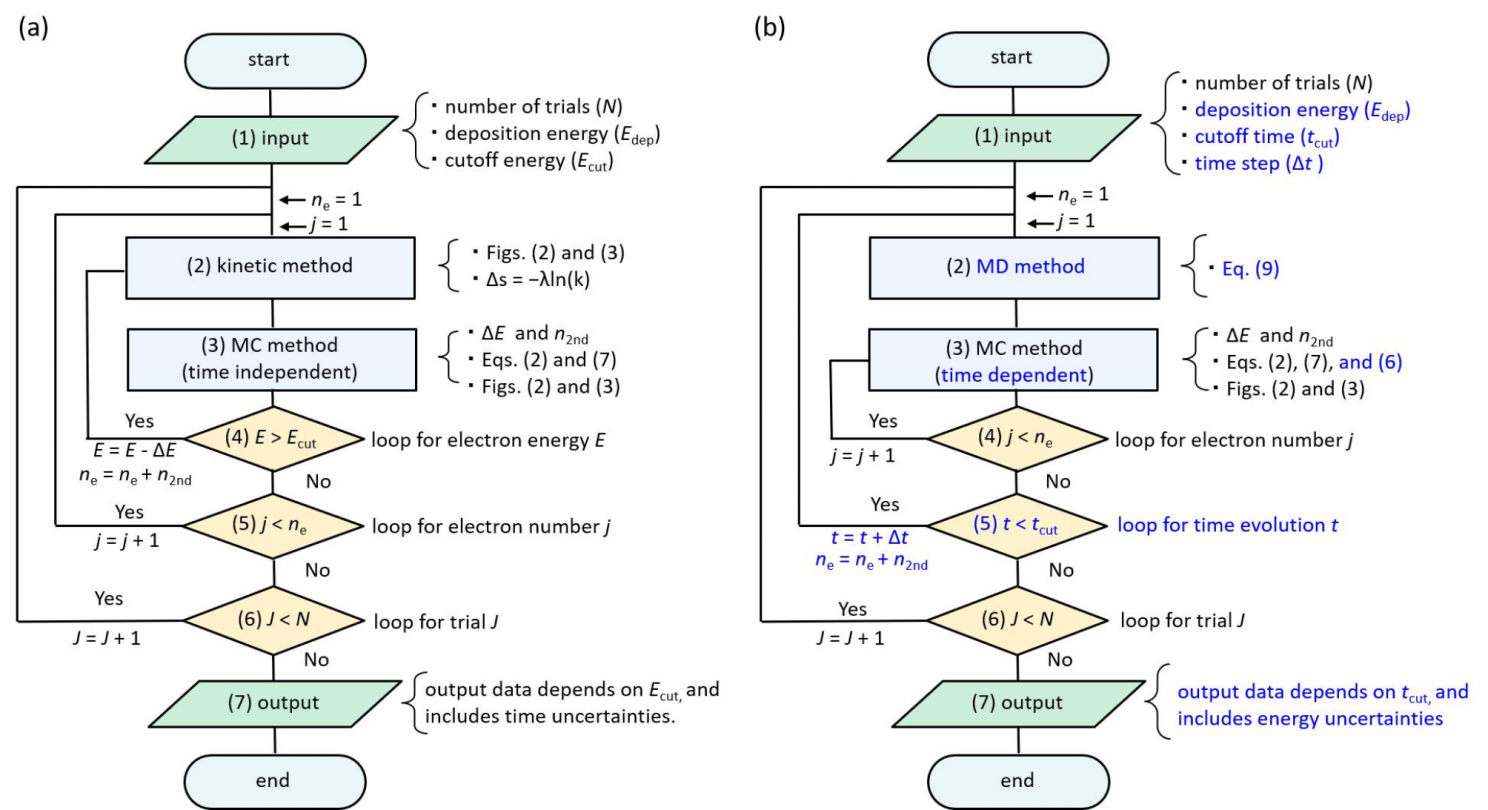
(**a**) Flowchart of conventional Monte Carlo codes (MCCs) for simulating an ejected electron generated by water photolysis or radiolysis, where Δ*E* is the energy loss. (**b**) Flowchart of dmcc_phys for simulating an ejected electron generated by water photolysis or radiolysis. Here, the initial kinetic energy of the ejected electron equals the deposition energy *E*_dep_. The velocity vector was determined using the Monte Carlo (MC) and molecular dynamics (MD) methods. MD allows electron relocalisation.

 This subsection shows a flowchart of conventional MCCs and dmcc_phys for multi-body chemical species spur formation to clarify the different calculation algorithms between the MCCs and dmcc_phys. Figure 5(a) shows a flowchart of conventional MCCs^1,26–35^, (1) where the inputs to the MCC are the number of trials (*N*), the deposition energy (*E*_dep_) and electron cut-off energy (*E*_cut_). The initial kinetic energy of the initial secondary electron was determined with *E*_dep_ − *I*_e_ (ionisation energy; 10.9 eV)^57^. (2) In the kinetic method, an electron is transported by Δ*s* = −*λ*ln(*k*).^26^ (3) In the MC method, the energy loss Δ*E* of the electrons and the number of generated electrons *n*_2nd_ are obtained. (4) Processes (2) and (3) are repeated until the initial secondary electron energy reaches the cut-off energy. (5) Then, for the additional secondary electron generated, repeat steps (2)–(4) in the same manner as for the initial secondary electrons. (6) Once these calculations are complete, move on to the next trial, *J* = *J* + 1. (7) All calculations are completed when the statistical uncertainties in the results are sufficiently small. In a conventional MCC, the calculated results depend on the cut-off energy. Therefore, the calculated results include time uncertainties due to energy identification.

Figure 5(b) shows the flowchart of our code (1), where the inputs to our code are the number of trials (*N*), the deposition energy (*E*_dep_), the cut-off time (*t*_cut_) of the calculation and the time step Δ*t* (1 attosecond). (2) In the MD method, the dynamic behaviours of the initial and additional secondary electrons are simultaneously solved for each time step Δ*t* following Eq. (9). (3) In the time-dependent MC method, electron–water collisions are determined by Eq. (6). If a collision occurs, the electron energy loss Δ*E* and the number of generated electrons *n*_2nd_ are obtained. (4) Process (3) is repeated for the number of *n*_2nd_. (5) After processes (2)–(4) are completed, we move on to the next time, *t* = *t* + Δ*t*. Processes (2)–(5) are repeated until the cut-off time is reached. (6) Once these calculations are complete, move on to the next trial, *J* = *J* + 1. (7) All calculations are completed when the statistical uncertainty of the results is sufficiently small. In our code, the calculated result depends on the cut-off time. Therefore, the results include energy uncertainties due to time identification.

## Results and discussions

First, we present the calculation results of electron spatial and collision frequency distributions at 22, 25, 27 and 30 eV deposition energies. Second, we show the energy dependence of the mean collision frequency and spur radius at 20–30 eV deposition energies. From these results, we finally discuss the formation mechanisms of clustered DNA damage.

### Electron spatial distribution

When high-energy electron transport deposits 20–30 eV energy in water, the ejected secondary electrons can immediately induce ionisation or electronic excitation. After induction, the secondary electrons become thermalised–delocalised and relocalised by molecular excitations and elastic scattering. In our previous study^47^, we demonstrated water photolysis at a deposition energy of 12.4 eV, indicating that the inter-molecular vibration mode is preferentially responsible for electron delocalisation and pre-hydration, whereas the rotation mode and elastic scattering are responsible for electron thermalisation and hydration. The simulation results indicated that e^−^ _aq_ forms after 300 fs or more at a deposition energy of 12.4 eV^47^. However, out latest study found that the thermalisation time increases as the deposition energy increases, and the time is ~ 500 fs at deposition energies of 17–19 eV^48^. In this study, the electron cut-off time was 500 fs. Under these conditions, we confirm that the electron-energy distributions sufficiently approach the Maxwellian of 300 K.

**Fig. 6:**
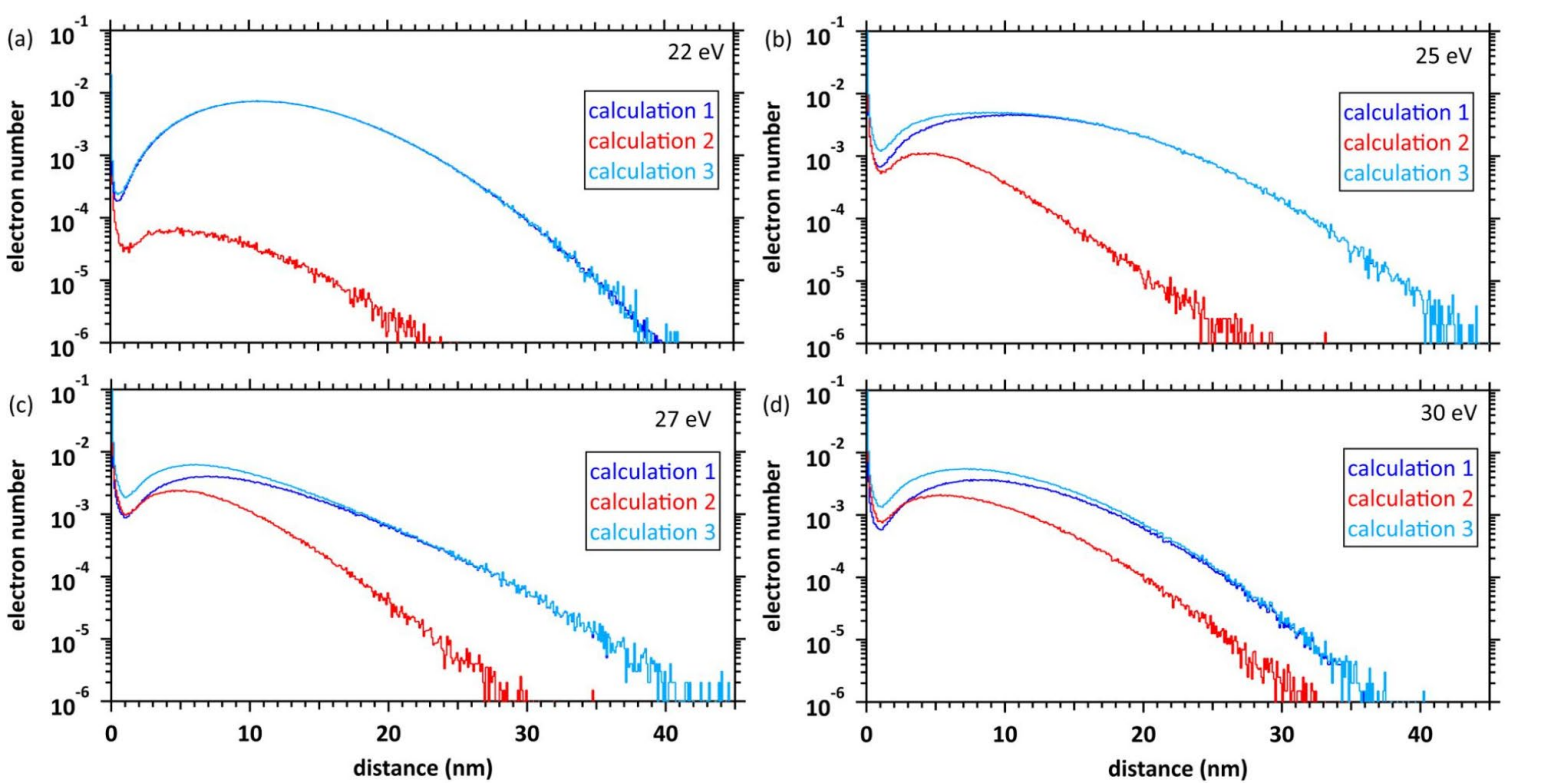
Spatial distributions of electron numbers at deposition energies of (**a**) 22, (**b**) 25, (**c**) 27 and (**d**) 30 eV at 500 fs. Calculation 1 shows the distribution of only the initial secondary electrons. Calculation 2 shows the distribution of additional secondary electrons. Calculation 3 shows the distribution of all electrons. The horizontal axis represents the relative distance from the ionic core of each parent cation. The distribution results are shown as spherical coordinates with a spatial mesh Δ*r* = 0.1 nm. All-solid angle meshes ΔΩ in the Δ*r* are integrated.

**Fig. 7:**
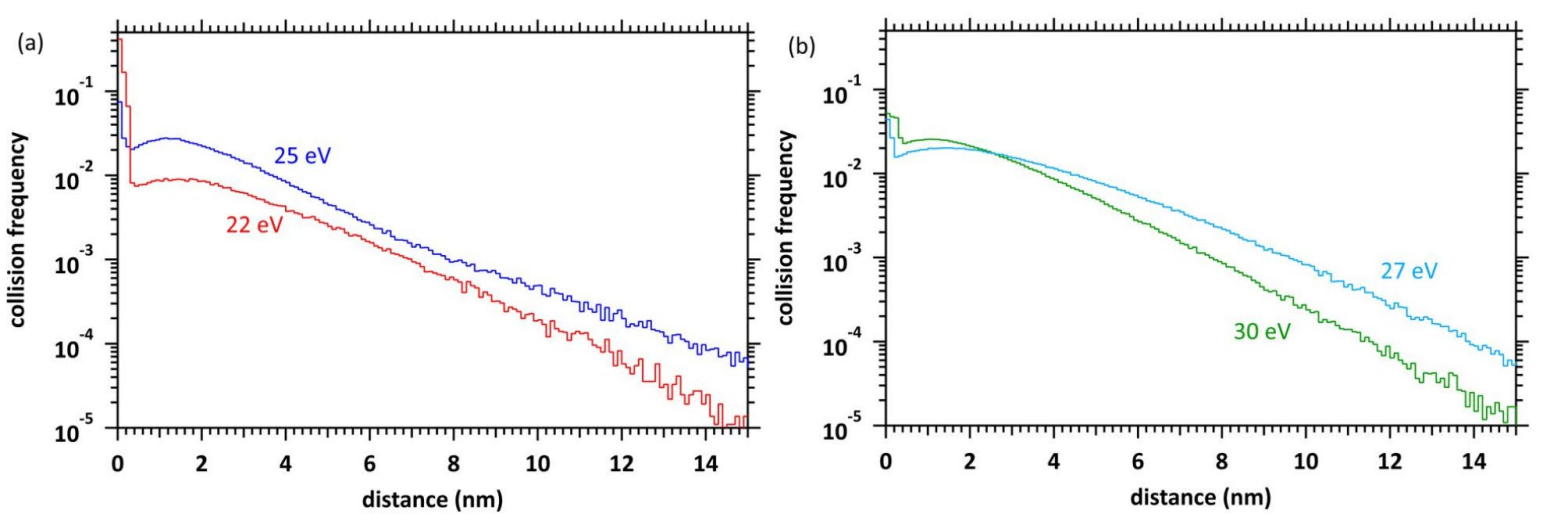
(**a**) Collision frequency distribution of ionisation plus electronic excitation at 22 and 25 eV deposition energies. (**b**) Collision frequency distribution of ionization plus electronic excitation at 27 and 30 eV deposition energies. The distribution results are shown as spherical coordinates with a spatial mesh Δ*r* = 0.1 nm. All-solid angle meshes ΔΩ in the Δ*r* are integrated.

Figure 6a–d depict the calculated results of the electron spatial distributions at 500 fs at 22, 25, 27 and 30 eV deposition energies, respectively. Calculation 1 in Fig. 6 shows the spatial distribution of the initial secondary electrons ejected by the initial energy deposition (blue line). Calculation 2 shows the spatial distribution of the additional secondary electrons newly produced by the collision of the initial secondary electrons (red line). Calculation 3 shows the spatial distribution of the sum of all electrons (light blue line). The distance on the horizontal axis indicates the relative distances between the initial electron and its parent ion, and between the additional electron and its parent ion. The positions of initial and additional ionisation are different. To indicate the absolute positions of the initial and additional secondary electrons separately or together, we need the support of Fig. 7 (next paragraph) which will provides information on the position of the additional secondary electron generation. The horizontal axis represents the relative distance from the ionic core of each parent cation. At 22 eV deposition energy, the number of additional secondary electrons is low (calculation 2 in Fig. 6a). Most additional secondary electrons are distributed in a very narrow region within 1 nm of the ionic core of the parent cations, indicating that they relocalise to the parent cations. Although some initial secondary electrons also relocalised to the parent cations, most delocalise to ~ 10 nm, forming a spur (calculation 1 in Fig. 6a). Production yields of additional secondary electrons gradually increase as the deposition energy increases above 25 eV. The electrons delocalise to ~ 5 nm from the ionic core of the new parent cations, whereas the relocalisation of those electrons decreases (calculation 2 in Fig. 6a–d). The initial secondary electrons delocalise to ~ 8 nm from the ionic core of parent cations above 25 eV (calculation 1 in Fig. 6b). In other words, when the deposition energy is above 25 eV, a multi-body chemical species spur forms and the spur radius decreases above 25 eV.

### Collision frequency distribution

Figure 7(a) and (b) show the collision frequency distributions at deposition energies of 22 and 25 eV and 27 and 30 eV, respectively. The collision frequency considerably increases in the region of a few Å at 22 eV deposition energy (Fig. 7(a)). Secondary electrons are ejected from the bottom of the potential assumed by our model with kinetic energy corresponding to the deposition energy (Fig. 4a). The potential energy is still sufficiently deep in the region of a few Å. Therefore, it can be interpreted that the cation potential before the electron deceleration induces multiple collisions in a single water molecule. Chemical species, such as 2H_3_O^+^, ^•^O^•^ and 2e^−^, might be produced by double proton transfer via H_2_O^2+^ induced by multiple collisions in a single water molecule. However, from Fig. 6(a) at 22 eV deposition energy, initial or additional secondary electrons are more likely to relocate to the parent cation when multiple collisions are induced. As the deposition energy is further increased, the rate of multiple collisions decreases because the time that electrons present in the molecule is shortened, and additional chemical species are generated in the region of a few nm from the parent cations, indicating multi-body chemical species spur formation.

### Spur radius

**Fig. 8:**
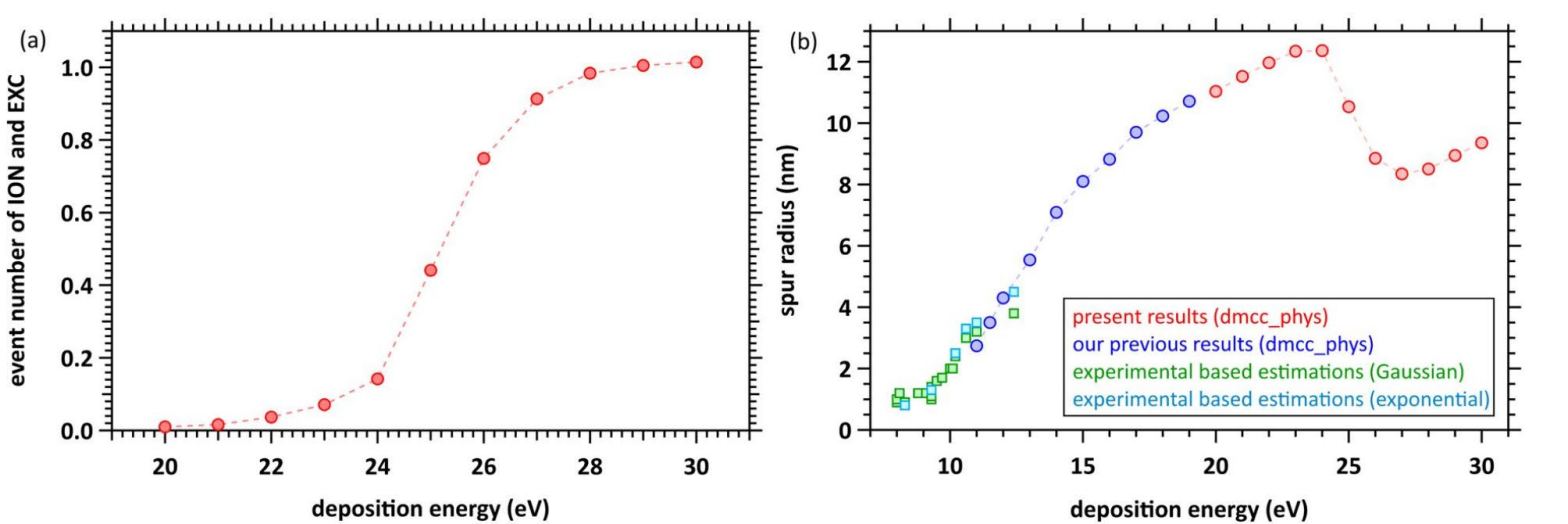
(**a**) Event number of ionisation and electronic excitation in the deposition energy region from 20 to 30 eV. (**b**) Spur radius of electrons in the deposition energy region from 8 to 30 eV. ☐; experimentally based estimation values^11–16^, 〇; our works (present and previous results^48^).

In our latest study^48^, we calculated the spur radius at deposition energies of 11–19 eV. From these calculated results and the experimentally based estimation values (8–12.4 eV) in the previous study^11–16^, we indicated that the spur radius generally increases linearly in the energy range. In this study, we first present the mean collision frequencies of the ionisations and electronic excitations induced by the initial secondary electrons in Fig. 8(a). Water is most likely to absorb the energy of 21.4 eV (Fig. 2(b)), and multiple collisions are sometimes induced by the induction of collective excitation at a deposition energy of 22 eV (Fig. 6(a) and 7(a)). However, additional ionisation or electronic excitations are gradually induced at several nm from the origin (Fig. 6(b) and 7(a)) above 25 eV deposition energy, and the initial secondary electrons finally delocalised to ~ 8 nm with many inductions of elastic scattering and molecular excitations (Fig. 6(b)). Here, diffuse band excitation with a mean transition energy of 14.1 eV (corresponding to the deposition energy of 25 eV minus the ionisation energy of 10.9 eV) can be induced, as described in the subsection (cross-section) of Methods (Fig. 2(c)). Our latest paper^48^ shows that electron relocalisation is small in diffuse band excitation induction, and electron delocalisation primarily produces H_3_O^+^, ^•^OH and e^−^. As the deposition energy reaches 30 eV, at least one additional ionisation is induced, indicating that a multi-body chemical species spur is formed.

Figure 8b shows the spur radius in the energy range of 8–30 eV. The present results for 20–30 eV deposition energies were estimated from calculation 1 in Fig. 6. The experimentally based estimation values^11–16^ and our previous results^48^ are also shown in the energy region of 8–12.4 eV and 11–19 eV, respectively. Our results show that the spur radius reaches a maximum of ~ 12 nm at 24 eV. A further increase in deposition energy induces diffuse band excitation; consequently, the spur radius decreases to ~ 8 nm in the 24–27 eV energy region. One additional ionisation or electronic excitation is induced at approximately 30 eV, resulting in spurs of multi-body chemical species.

**Fig. 9:**
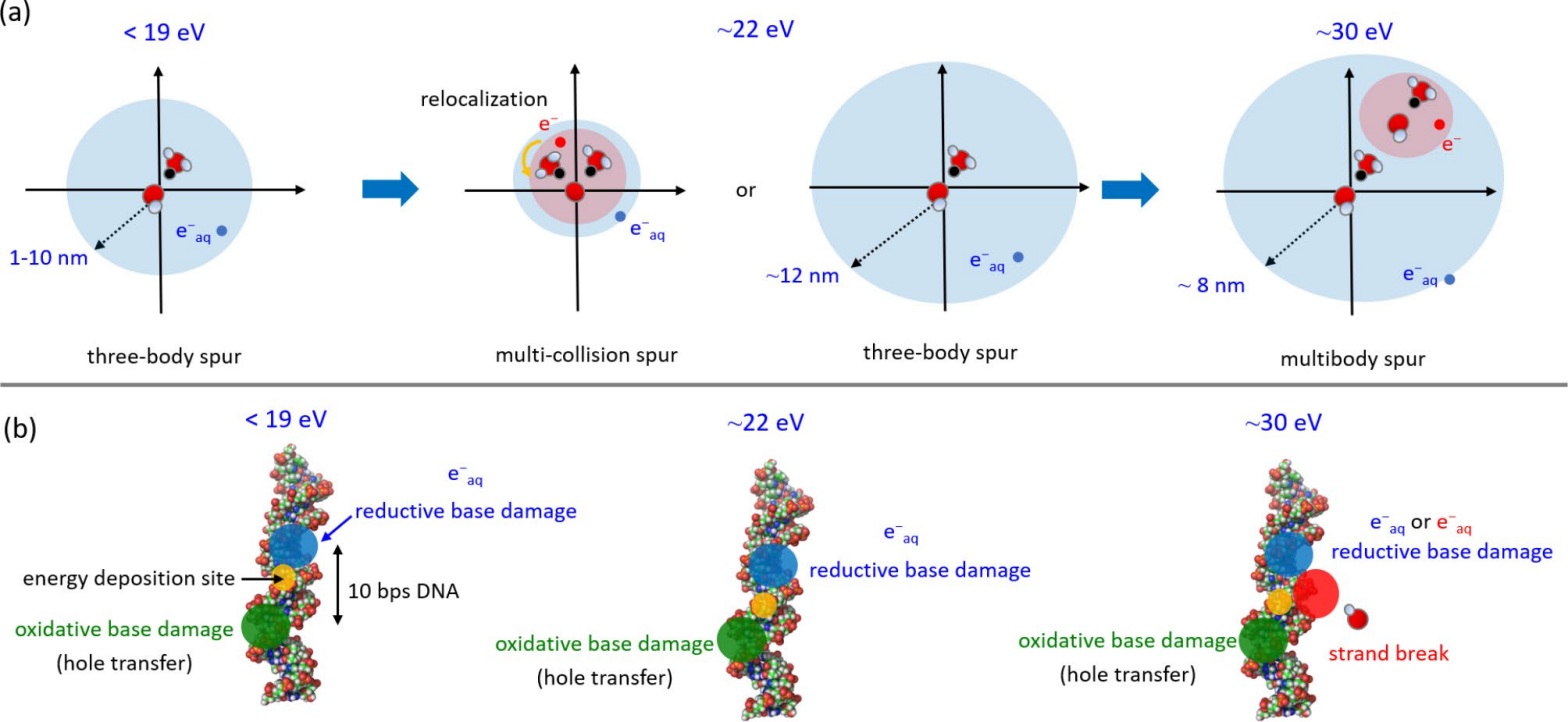
(**a**) Illustration of the summary of this study. (**b**) Prediction of clustered DNA damage.

This work clarifies how the chemical species formed in the spurs change with increasing deposition energy. Figure 9(a) summarises the present results. A three-body spur is formed when the deposition energy is < 19 eV. A multi-collision spur is sometimes formed, and initial or additional secondary electrons are relocalised above 22 eV. When the deposition energy reaches 30 eV, multi-body spur formation is completed.

### DNA damage

 The damage sites induced by a spur within a 10 bps (i.e., 3.4 nm) are clustered DNA which are thought to be a major genetic effect, such as cell death or mutation induction. Even when the size of the clustering events is beyond 3.4 nm, they are categorized as “complex types of DNA damage” possibly causing the deleterious effect of cells depending on their complexities^68–70^. Here, we discuss the clustered DNA damage formation mechanism from our calculated results. Our study assumed that the initial secondary electron was emitted from DNA by deposition energy via high-energy electron transport in an aqueous DNA solution. Figure 9(b) schematically illustrates clustered DNA damage formation in a 10 base-pair segment for three energy deposition cases predicted by our results. The damage complexity is discussed based on our results integrated with previous experimental insights of the direct DNA oxidation by high-energy electrons^71–80^ as follows.

1. *The deposition energy < 19 eV.* When the deposition energy of DNA from the high-energy electrons is below 19 eV, a produced secondary electron is decelerated and finally formed e^−^ _aq_ distributes within 10 nm around the ionised DNA as revealed by present study. The positive charge generated on DNA is likely to move on guanine via hole transfer^71–73^ to form a guanine cation^74^, and then an oxidative base lesion, such as 8-oxo-guanine (8-oxo-G), is formed after deprotonation from the cation. The e^−^ _aq_ are thermally diffused, even though the frequency of e^−^ _aq_ arriving within 10 bps DNA would not be high. Once a e^−^ _aq_ reacts with the ionised DNA within 10 bps, a reductive base lesion, such as dihydrothymine (DHT) via thymine anion^74^, takes hold. Thus, clustered damage consisting of one oxidative base lesion (direct energy deposition to DNA) and one reductive base lesion (indirect action of e^−^ _aq_).
2. *The deposition energy ~ 22 eV.* The ELF of water is most likely to absorb energy around 21.4 eV through radiation transport (Fig. 2(b)). The deposition energy on DNA causes multiple localised collisions, as shown in the multi-collision spur (~ 22 eV) in Fig. 7(a). If either the initial or additionally produced secondary electron relocalises to their parent cations, the electron may be recaptured the cation. On the other hand, if both electrons escape from the Coulomb field of the cations, they could be hydrated and thermally diffuse to interact with a DNA base with much higher frequency than the case of the deposition energy < 19 eV as described above. Consequently, these events may produce a more complex combination of an oxidative and one (or two) reductive base lesion(s). This model well consistent with previously experimental reports using fully hydrate plasmid DNA films. The pyrimidine base lesions, such as DHT, produced by γ-rays are 1.3 times larger than that for oxidative base lesion, such as 8-oxo-G^75^. This ratio is significantly enhanced to be over 2-fold for X-ray^76^ or He^2+^ ion^77^ and C^5+,6+^ and Ne^8+,10+^ ion irradiations^78^.
3. *The deposition energy > 25 eV.* When the deposition energy is above 25 eV, chemical species (^•^OH, e^−^ _aq_ and H_3_O^+^) are densely produced in the DNA neighbourhood (Figs. 6 and 7). These chemical species are thermally diffused and facilitate a multibody spur reaction around DNA. When ^•^OH and e^−^ _aq_ approach within 10 bps DNA, they react with the DNA backbone and base, respectively^74^, resulting in single-strand breaks (SSBs) and reductive base lesions. Thus, a highly clustered damage site consisting of the oxidative base lesion, the reductive base lesion and the SSB (^•^OH action) would be formed within 10 bps DNA. Experimental evidences are scarce because of technical difficulties to detect base lesions proximately arising a SSB^79^. Recently, enhanced green fluorescent protein expressing plasmid DNA possessing SSBs were used to assess the SSB repair efficiency in human cells^80^. They showed that repair rate of the SSBs produced by X-rays were significantly lower than those produced by restriction enzymes, suggesting that the base lesions proximately positioned at the SSB termini compromise the access of DNA repair enzymes.

These results strongly indicate that the types and sites of clustered or complex DNA damage strongly depend on the amount of the initial deposition energy. As the energy deposited on DNA increases, DNA damage becomes more complex. On the other hand, as shown in Fig. 2b, ELF decreases with increasing energy, indicating that the higher energy deposition on DNA is reduced its frequency. Hence the damage complexity and its frequency have a compensatory relationship.

Even though these estimations were based on an assumption that e^−^ _aq_ and ^•^OH induce damage with a same probability by their reactions with DNA, the results for SSBs, oxidative or reductive base damage are in excellent agreement with quantitative findings obtained in previous experiments where the yield of damage caused by an ^•^OH, such as 8-oxo-G or SSB, was observed at approximately the same yields as reductive damage, such as dihydrothymine^76^. Furthermore, the results also showed that clusters containing the oxidative base damage are generated at approximately the same yield as clusters containing reductive base damage^76^, which successfully support our assumption that the e^−^ _aq_ and ^•^OH arising proximately each other create a clustered DNA damage. Thus, the present study was the first to show that the nanoscale localization of radiation products of water decomposition causes clustering of DNA damage.

### Future plan

We would be interesting to analyse experimental data for intermolecular Coulombic decay^81^ to advance electron ejection algorithm triggered by ionisation of valence band electron although the molecular conformational changes cannot be calculated. In addition to the dmcc_phys^40–48^ used in this study, the original chemical code (dynamic MCC for the chemical process; dmcc_chem) was developed^82^. dmcc_chem considers the dielectric response for calculating the diffusion and reaction of the chemical species generated in water. Recently, we calculated electronic states of poly (CG) DNA by first-principles calculation using OpenMX^83^. In the near future, dmcc_phys, dmcc_chem and OpenMX^84^ will be connected to demonstrate complexity of DNA damage formation.

## Conclusion

The evaluation of spur-radius dependence on deposition energy by radiations or lasers is very important in radiation chemistry. This is because the reaction time of the produced chemical species strongly depends on the spur radius^11–16^. In other words, the reaction time strongly depends on the deposition energy. Whereas the previous studies^47,48^ focused on the formation of three-body chemical species, the present study focused on the 20–30 eV deposition energies that produce multi-body chemical species. When the energy deposited to water changes from 11 to 30 eV, the reaction time and reaction pathway of the chemical species also change. The present results provide new significant insights to the radiation chemistry.

The collision frequency distribution for water radiolysis was shown in our previous study^46^. However, the deposition energy dependence was not indicated. The present results for the dependence indicated that multiple collisions were induced when the deposition energy was around 22 eV and that multi-body spurs were formed when the energy exceeded 25 eV. Therefore, the energy deposition above 25 eV makes it possible to form the clustered DNA damage, which has important biological significance. These findings provide new significant insights into the radiation biophysics.

Yields for single-strand breaks and double-strand breaks of DNA have been measured by many experiments. Recently, although the yields of the clustered DNA damage have also been measured^85^, it is extremely difficult to detect redox lesions generated in the vicinity of the strand break. The yields of various types of the clustered DNA damage with the redox lesions could already be evaluated by computer simulations^85^. However, the simulation includes a bold model with simplified direct and indirect effects^85^. Currently, a reliable understanding of the formation mechanism of the clustered damage is desired over the model simulation. Our results provide a powerful clue to deeply understand the formation mechanism. Advanced quantitative evaluation of the damage yield will require a connection to the chemical code^70^. The connection will be realised in the near future.

## Data Availability

The data supporting the findings of this study are available from the corresponding author upon reasonable request.
